# Current Challenges of Digital Health Interventions in Pakistan: Mixed Methods Analysis

**DOI:** 10.2196/21691

**Published:** 2020-09-03

**Authors:** Abdul Momin Kazi, Saad Ahmed Qazi, Nazia Ahsan, Sadori Khawaja, Fareeha Sameen, Muhammad Saqib, Muhammad Ayub Khan Mughal, Zabin Wajidali, Sikander Ali, Rao Moueed Ahmed, Hussain Kalimuddin, Yasir Rauf, Fatima Mahmood, Saad Zafar, Tufail Ahmad Abbasi, Khalil-Ur-Rahmen Khoumbati, Munir A Abbasi, Lampros K Stergioulas

**Affiliations:** 1 Department of Pediatrics and Child Health Aga Khan University Karachi Pakistan; 2 Department of Electrical Engineering NED University of Engineering and Technology Karachi Pakistan; 3 Neurocomputation Lab National Center of Artificial Intelligence Karachi Pakistan; 4 NED University of Engineering and Technology Karachi Pakistan; 5 Biomedical Department NED University of Engineering and Technology Karachi Pakistan; 6 Riphah International University Islamabad Pakistan; 7 University of Sindh Jamshoro Pakistan; 8 Surrey Business School University of Surrey Guildford United Kingdom

**Keywords:** digital health, eHealth, LMICs, mHealth, Pakistan, SWOT, telehealth

## Abstract

**Background:**

Digital health is well-positioned in low and middle-income countries (LMICs) to revolutionize health care due, in part, to increasing mobile phone access and internet connectivity. This paper evaluates the underlying factors that can potentially facilitate or hinder the progress of digital health in Pakistan.

**Objective:**

The objective of this study is to identify the current digital health projects and studies being carried out in Pakistan, as well as the key stakeholders involved in these initiatives. We aim to follow a mixed-methods strategy and to evaluate these projects and studies through a strengths, weaknesses, opportunities, and threats (SWOT) analysis to identify the internal and external factors that can potentially facilitate or hinder the progress of digital health in Pakistan.

**Methods:**

This study aims to evaluate digital health projects carried out in the last 5 years in Pakistan with mixed methods. The qualitative and quantitative data obtained from field surveys were categorized according to the World Health Organization’s (WHO) recommended building blocks for health systems research, and the data were analyzed using a SWOT analysis strategy.

**Results:**

Of the digital health projects carried out in the last 5 years in Pakistan, 51 are studied. Of these projects, 46% (23/51) used technology for conducting research, 30% (15/51) used technology for implementation, and 12% (6/51) used technology for app development. The health domains targeted were general health (23/51, 46%), immunization (13/51, 26%), and diagnostics (5/51, 10%). Smartphones and devices were used in 55% (28/51) of the interventions, and 59% (30/51) of projects included plans for scaling up. Artificial intelligence (AI) or machine learning (ML) was used in 31% (16/51) of projects, and 74% (38/51) of interventions were being evaluated. The barriers faced by developers during the implementation phase included the populations’ inability to use the technology or mobile phones in 21% (11/51) of projects, costs in 16% (8/51) of projects, and privacy concerns in 12% (6/51) of projects.

**Conclusions:**

We conclude that while digital health has a promising future in Pakistan, it is still in its infancy at the time of this study. However, due to the coronavirus disease 2019 (COVID-19) pandemic, there is an increase in demand for digital health and implementation of health outcomes following global social distancing protocols, especially in LMICs. Hence, there is a need for active involvement by public and private organizations to regulate, mobilize, and expand the digital health sector for the improvement of health care systems in countries.

## Introduction

The World Health Organization (WHO) addresses digital health as a global strategy in 2020-2024, and its digital health policy draft defines digital health as “the field of knowledge and practice associated with the development and use of digital technologies to improve health” [1]. It is a broad umbrella term encompassing mobile health (mHealth), eHealth, telemedicine, and advanced computing sciences like genomics, artificial intelligence (AI), and big data [2]. Digital health is a rapidly growing industry that, according to some estimates, is expected to be valued at US $504.4 billion by the end of 2025 [3]. It is being viewed as an accessible and affordable solution for people who do not have access to the traditional health system, and an important tool in achieving sustainable development goals [4].

Digital technologies like artificial intelligence (AI) and machine learning (ML) are an integral part of many businesses and companies in the developed world. They are a driving force in multimillion-dollar industries, such as automotive manufacturers, who rely on AI to, for example, predict when cars might need repair to ensure the safety of passengers [5]; the banking sector, where AI is applied to detect fraudulent transactions [6]; and Silicon Valley giants like Google, Facebook, and others, who have formed a consortium for conducting research intended to improve the understanding of AI technologies for the improved welfare of society [7]. The health sector is also embracing the digital revolution; digital health technology in developed countries is being employed in major aspects of the health care process, including consultation, diagnosis, treatment, monitoring, patient education, behavioral modification, and medication adherence [8-11]. In the fast-growing field of health AI, a recent study in Japan used an AI-based diagnostic system that demonstrated a higher diagnostic accuracy for esophageal carcinoma than those from conventional methods [12]. A retrospective study conducted to evaluate the accuracy of AI in predicting the mammograms of biopsy-proven breast cancer patients showed that AI outperformed all of the human readers [13]. Similar studies conducted on dermatological lesions using deep convolutional neural networks have shown that the ability of AI to classify malignant and nonmalignant conditions correctly is comparable to that of board-certified dermatologists [14].

A meta-analysis evaluating the impact of digital health interventions on noncommunicable diseases (NCDs) showed that the interventions significantly reduced the occurrence of cardiovascular outcomes such as stroke and myocardial infarction through positive behavior change theory [15]. mHealth-based interventions such as MyAirCoach (Asthma UK) are helping patients achieve better control of asthma-related symptoms by educating them on proper inhaler use and providing personalized treatment plans in coordination with health care providers in cases of exacerbations [16]. As seen in relevant randomized controlled trials, this intervention is part of an initiative to digitalize asthma care in the National Health Service (NHS) in the United Kingdom [16-18]. In the United States, over 61% of health care institutions provide telemedicine services, which are covered by insurance policies and Medicare programs [19,20]. According to estimates, telemedicine services helped the US Department of Veterans Affairs reduce its hospitalization for mental health diseases by over 40% in 2012 [21,22].

The expansion of mobile and wireless technologies offers an unprecedented opportunity for global health delivery in low and middle-income countries (LMICs). Digital health innovations are addressing issues such as maternal, newborn, and child health; low immunization coverage; lack of access to life-saving medications; infectious disease outbreaks; and the increasing burden of NCDs [23,24]. Sub-Saharan African countries are using an SMS text messaging technology project, SMS for Life, to ensure accurate reporting of real-time facility stock data to reduce antimalarial drug stock-outs [25,26]. India is using digital health tools to combat tuberculosis pandemics through eCompliance, which helps to monitor patients in real-time, ensure better medication adherence, and decrease treatment default rates [27-29]. Bangladesh is storing its health information data in a common data repository called the District Health Information Software 2 (DHIS2, Health Information Systems Programme), which allows for real-time monitoring, accurate localization of under-resourced areas, and better resource allocation [30,31].

In Pakistan, the doctor-to-patient ratio is close to 0.83 physicians per 1000 individuals in the population. Digital health interventions are being designed to address various health care needs. Several SMS-based interventions are being used to improve medication compliance in patients with NCDs [32], and telemedicine tools are being used to educate patients and to keep health care professionals abreast of medical advancements [33]. Moreover, as many female doctors leave clinical practice due to household and childcare responsibilities, telemedicine initiatives such as eDoctor (SE Software Technologies) and Sehat Kahani (Grocode.io) enable them to conduct their medical practices remotely via online patient consultation through a telehealth platform [34-36]. Another intervention, a mobile app called Teeku (Aga Khan University) aimed at helping vaccinators record immunization data, generated reliable data for better monitoring and improved the coverage of expanded program on immunization (EPI) vaccines such as the pentavalent and pneumococcal conjugate vaccine [37]. Outbreak investigation and surveillance is another relevant public health domain that can be improved with digital health interventions. In 2011, after a particularly severe Dengue outbreak, a GPS-enabled mobile application called the Dengue Activity Tracking System (Punjab Information Technology Board) was developed to track suspected and confirmed cases of dengue [38]. In 2 recent epidemics of the extensively drug-resistant typhoid and human immunodeficiency virus (HIV) in the province of Sindh, geospatial mapping helped considerably with identifying the root causes of the outbreaks and with isolating cases and their spreads [39,40].

These aforementioned programs are just a few examples of successful digital health interventions carried out in Pakistan, despite limited resources available due to the financial constraints of the country. Mobile app solutions and social media have been shown to be quite effective in various programs worldwide, but there is limited data from LMICs on the use of emerging technologies in improving health care services. In this study, we propose to identify the current digital health projects being carried out in Pakistan and the key stakeholders involved in these initiatives. Further, we evaluate these projects and studies through strengths, weaknesses, opportunities, and threats (SWOT) analysis to identify the internal and external factors which can potentially facilitate or hinder the progress of digital health in Pakistan.

This will enable us to highlight specific challenges and areas that require digital health, to assess gaps in the current system, and to identify the roles of health care providers, technology partners, public and private partners, and policymakers in creating an environment for digital health fraternity to sustain prosperity.

## Methods

### Identification of the Targeted Population

The main objective of this study is to investigate the major digital health interventions in Pakistan during the last 5 years. The target population includes individuals or teams who have used technology-based interventions in the field of digital health.

### Research Design

This study is conducted through mixed methods [41,42]. The data were gathered following the conceptual framework of Arksey and Mallor [43], categorized according to the WHO’s recommended building blocks for health systems research and analyzed using the SWOT analysis strategy [44].

**Figure 1:**
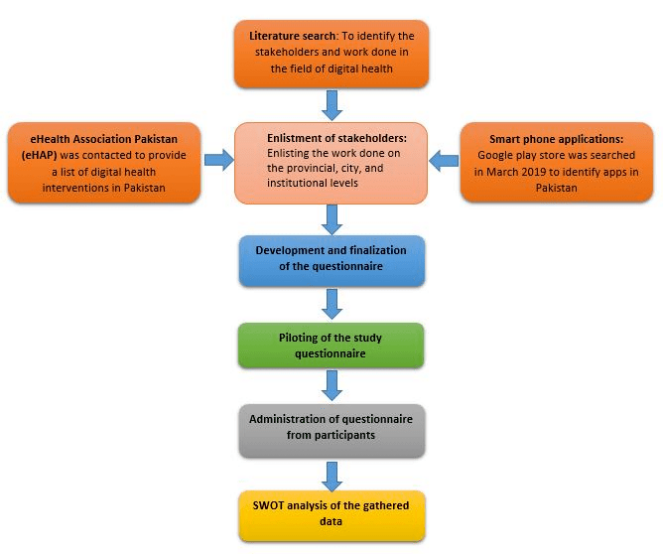
Study flow and major components of the study methodology.

Figure 1 describes the flow of the study and the major components of the study methodology. The study was divided into 3 phases: Phase 1 was related to a literature search to identify relevant authors, papers, and interventions; Phase 2 was related to questionnaire development, piloting, and data collection using a mixed-methods approach; Phase 3 involved the SWOT analysis of the collected data.

### Inclusion and Exclusion Criteria

All studies in digital health conducted in Pakistan over the last 5 years were included.

Projects, innovations, and interventions that were completed more than 5 years ago in Pakistan were excluded from our study.

### Research Population

An electronic, systematic, in-depth literature search was conducted in March 2019 using PubMed, Google Scholar, Web of Science, J.Store, Academia, and Pak Medinet. The key medical subject headings (MeSH) terms used for these databases included “mHealth” [MeSH Terms] AND “Pakistan” [MeSH Terms], “digital health” [MeSH Terms], OR “telehealth” [MeSH Terms], OR “Mobile phones” [MeSH Terms], OR “Cell Phone”[MeSH Terms] OR “Mobile Applications”[MeSH Terms] OR “Text Messaging”[MeSH Terms] OR “Telemedicine”[MeSH Terms] AND “Pakistan”[MeSH Terms].

A search strategy similar to that of a systematic review was used to identify the maximum number of interventions, projects, studies, or smartphone applications that had been carried out in the field of digital health in Pakistan. The search for published studies was not limited to any particular study design; all types of study designs were included.

In addition to published work, applications uploaded on Google Play store were also evaluated and shortlisted for further analysis. Google Play was thoroughly searched, as 95% of the smartphones used in Pakistan are Android-based, and usually, the apps available in other app stores are already on this platform [45,46]. We also contacted the eHealth Association of Pakistan (eHAP) for information on current digital health studies. Studies were excluded from the review if the project was not based in Pakistan, did not explicitly involve digital health interventions (eg, telemedicine, other types of eHealth, or used other telecommunication technologies such computers, internet, or email), or examined only feasibility or acceptance of eHealth in society.

The data collected was then independently assessed for inclusion and eligibility. Any duplicated titles were removed, and the final list of published studies, projects, innovations, and smartphone applications was then imported on to a Microsoft Excel file (Multimedia Appendix 1). The projects were divided into different categories, including telemedicine projects, mobile apps, studies published in the last five years, as well as their authors, provinces, and institutions that the authors were affiliated with. This system of organization eased the process of approaching respondents.

### Identification of Stakeholders and Mapping

The project team then made efforts to contact the stakeholders of the projects identified in the literature search and brief them regarding the study objectives. This data was used for stakeholder engagement activities, including questionnaire surveys and co-design workshops. The co-design workshops were carried out in various cities in Pakistan. For this study, 51 stakeholders were approached. Each participant or stakeholder represented an individual study or intervention. The organizing team members met the stakeholders, both in-person and online, to ensure streamlined communication with everyone involved. This also provided information regarding other unreported or unpublished technical innovations.

### Questionnaire Development and Administration of the Stakeholder Survey

A questionnaire with qualitative and quantitative sections was developed for data collection, which included both open-ended and close-ended questions. The questionnaire included sections on (1) the respondents’ details, (2) project details, (3) team details, (4) project technology, (5) project evaluation, and (5) ethics in digital health, which were captured in line with the WHO’s building blocks for health systems [44,47]. Pilot testing of the questionnaire was conducted initially to help us refine the questionnaire. The finalized study questionnaire was administered to the core project team members who had health and technology expertise.

### Data Analysis

A mixed-methods strategy was used for data analysis. The collected data was divided into 3 sets of analysis: quantitative, qualitative, and SWOT analysis. The quantitative data were analyzed in Matrix Laboratory (MATLAB R2015b 9.7, MathWorks) and SPSS (version 19.0, IBM Corp) to run basic frequencies, while the qualitative data were reviewed for familiarization, coding, and the generation of themes. The findings were presented as a descriptive summary and thematically analyzed according to the WHO’s defined health system building blocks [44,47]. The main frequencies of the quantitative data and the themes derived from the qualitative data were summarized and extracted into a table of 4 main groups: strengths, weaknesses, opportunities, and threats (SWOT).

SWOT analysis was performed to identify the internal factors (including the demographics of the researcher and the institute, scalability, sources of funding, and feasibility) and external factors (including the diseases targeted by the projects, technologies being used to carry out the intervention, monitoring and evaluation, and ethical challenges) that influenced the implementation of the project or study [48]. SWOT analysis also provided insight into the often complex relationships between the various factors and aspects necessary to create sustainable improvements. Lastly, SWOT analysis was used to serve as a main strategic project planning tool to better inform decision makers in evaluating the impact of different digital health initiatives in Pakistan.

### Data Management

The finalized questionnaire was developed as a web-based application connected to a MySQL database server, hosted on a secured local cloud. The development of the web application was tested for correctness, timely data inputs, and secure data saving. The team administering the questionnaire was trained in 2 separate group sessions, in which the study methodology and design were explained, and each question and the associated probes were discussed in detail. This team was then divided to approach the stakeholders individually and administer the questionnaire through face-to-face, telephonic, and Skype interviews lasting 20-30 minutes, after obtaining the interviewees’ written consent. The responses were uploaded in real-time and stored anonymously using a coded respondent ID, and both quantitative and qualitative inputs were stored digitally.

### Ethical Considerations

The study protocol and associated study instruments, including the consent form, were approved by NED University of Engineering and Technology and Aga Khan University’s Ethics Review Board before the commencement of any study activities.

## Results

### Quantitative Results

Baseline information of the quantitative survey is shared in Table 1. Most of the 51 respondents were public sector (17/51, 34%) and private sector (17/51, 34%) employees working in health care organizations, followed by academia (9/51, 17%) and nongovernmental organizations (1/51, 2%). Of the 51 projects, 57% (29/51) commenced after 2016, 33% (17/51) commenced between 2011 and 2015, and 6% (3/51) commenced between 2006 and 2010. Regarding funding, 37% (19/51) of interventions were funded internally, followed by 29% (15/51) funded internationally and 16% (8/51) funded nationally. Of the applications designed, 56% (28/51) were close source and 14% (7/51) were open source.

**Table 1:** Baseline information of the digital health studies and projects in Pakistan (N=51).

Variables		n (%)
**Age (years)**		
	20-30	5 (10)
	31-40	31 (61)
	41-50	10 (20)
	>50	5 (9)
**Gender**		
	Male	41 (80)
	Female	10 (20)
**Field of expertise (n=47)**		
	Information technology	22 (47)
	Engineering	6 (13)
	Public health	6 (13)
	Digital health	9 (19)
	Others	4 (9)
**Department (n=44)**		
	Computer science and information technology	11 (25)
	Pediatrics	9 (21)
	Primary and secondary health care department	8 (18)
	Engineering	8 (18)
	Other	8 (18)
**Province/territory where the project is being conducted**		
	Sindh	32 (65)
	Punjab	9 (18)
	Federal capital	5 (10)
	Gilgit Baltistan	2 (4)
	Khyber Pakhtunkhwa	1 (2)
	Not answered	2(4)
**Developer of the application (n=43)**		
	University	16 (37)
	Private institute	12 (28)
	Semi-government institute	8 (19)
	Individual developer	7 (16)
**How do you define work on technology in health? (n=43)**		
	Digital health	19 (44)
	Technology in health	16 (37)
	General health	5 (12)
	Geospatial	2 (5)
	Device development	1 (2)
**Did you report the study/project dissemination? Select all that apply (n=31).**		
	Publications	12 (39)
	Websites/blogs	11 (35)
	Government and funding agency	5 (16)
	Not yet	3 (10)

Of the 51 interventions, 45% (23/51) were centered around research, 29% (15/51) on implementation, 12% (6/51) on application or software development, 4% (2/51) each on prototype or device development, and 2% (2/51) each for commercial projects and system development. Core teams consisted of professionals from different backgrounds; information technology (IT) professionals comprised 40% (21/51) of the core teams, followed by investigators (5/51, 10%), epidemiologists (5/51, 10%), students (4/51, 8%), administrators (3/51, 7%), health care professionals (3/51, 7%), field staff (3/51, 7%), electrical engineers (2/51, 4%), biomedical engineers (2/51, 4%), psychologists (2/51, 4%), and cell phone providers (1/51, 2%).

The health domains targeted by the interventions included general health (23/51, 46%), immunization (13/51, 26%), diagnostics (5/51, 10%), and mental health and behavioral change (3/51, 6%). The disease outcomes targeted were NCDs in 56% (29/51) of the interventions, infectious diseases in 33% (17/51) of the interventions, and mental health in 7% (4/51) of the interventions. In regard to target populations, 33% (17/51) of the interventions catered to the general population, 30% (15/51) were specifically for children and adolescents, and 20% (10/51) targeted adults only. The targeted population belonged to all the various socioeconomic classes, with 39% (20/51) belonging to the middle class, 38% (19/51) to lower socioeconomic class, and 23% (12/51) to upper socioeconomic class, as per the participants’ self-rating.

These projects are also using innovative ideas for their implementation: 30% (15/51) of the respondents reported using a new technique or method, 17% (9/51) implemented the technology in a novel way, and 15% (8/51) used a centralized system; 9% (5/51) of the interventions were low-cost and mobile app–based and another 9% (5/51) were based in remote areas with constraint settings.

Smartphones and devices were the most commonly used component of digital health, used in 55% (28/51) of the interventions; websites and portals were employed in 25% (13/51), telehealth was used in 9% (5/51), special devices like e-stethoscopes and e-ultrasounds were used in 9% (5/51) of the projects, and 3% (2/51) used feature mobile phones and functional phones. When asked about barriers encountered in study implementation, 21% (11/51) reported that the population could not use the technology or mobile phones. Cost was the second most common barrier, reported by 16% (8/51) of the responders, followed by privacy concerns, which were raised by 12% (6/51) of the responders.

At the time of the study, 37% (19/51) of projects were fully operational, 26% (13/51) were completed, and 15% (8/51) were in the process of being scaled up. In regard to the development stage of the technologies, 44% (22/51) of the technologies used in the interventions were fully launched, 34% (17/51) were in the pilot phase, 16% (8/51) were in the conceptual stage, and only 3% (2/51) of the technologies employed were regularized. Major barriers faced in implementing the project-specific technology were the cost of technology for 28% (14/51) of the projects, lack of skilled personnel for 18% (9/51), and internet connectivity issues for 16% (8/51). The major costs involved in most projects were equipment and infrastructure costs in 42% (21/51) of the projects, followed by human resource personnel for 23% (12/51) and field implementation for 18% (9/51). For storage, 73% (37/51) of projects used a local server for data storage while the rest (14/51, 27%) engaged cloud technology. MySQL was used in 66% (34/51) of projects as a server for the database, while 11% (6/51) used SQLite, 9% (5/51) used Firebase, and 9% (5/51) used Postgres Structure Query Language (PostgreSQL). Programming languages used include Java (15/51, 30%), C/C++ (12/51, 24%), PHP (10/51, 20%), Python and Java (4/51, 7% each), DOT Net and ROR (3/51, 6% each).

Users were required to undergo some training specific to the technology being used in 76% (39/51) of the interventions, and this training varied in terms of duration. The individuals and groups most likely to benefit from the interventions are health care providers (22/51, 44%), followed by global health organizations (17/51, 33%) and digital health administration (17/51, 33%). At the time of the survey, 74% (38/51) of the projects were being evaluated.

AI or ML is being used in 31% (16/51) of the projects, with most projects using TensorFlow (19/51, 38%), MATLAB (16/51, 31%), and General Architecture for Text Engineering (GATE; 8/51, 15%). Only 2% (1/51) of the projects were connected to a national database. Plans for scaling up were present in 59% (30/51) of the projects; 13% (7/51) were fully commercialized and 7% (4/51) were awaiting patent approval. Data were accessible to only the administrators in 76% (39/51) of the projects. Funding agencies and universities were given access to 5% (3/51) of the projects. All respondents agreed on having a data retention policy beyond the project duration. Ethics board approval before implementation was required for 51% (26/51) of the projects, 33% (17/51) required licensed software, and 6% (3/51) required approval from regulatory bodies before implementation. Written consent from participants was taken in 42% (21/51) of projects, 33% (17/51) utilized verbal consent, 7% (4/51) used electronic consent, 9% (5/51) each did not take consent or did not require it for the intervention.

### Qualitative Results

The 4 SWOT themes that were identified are the following: (1) project novelty, (2) technology constraint, (3) catalyzing culture, and (4) workforce training duration.

#### Project Novelty

The results of the study show that the research population, including professionals in IT, engineering, public health, and digital health across different provinces, prefer project innovation to be focused on the domains of telehealth, AI diagnostics, and the digitization of EPI data. At present, the projects are in different phases of development, such as exploratory, research, ideation, delivery, and others.

#### Technology Constraints

The professionals with diverse backgrounds (which include individuals from IT, engineering, public health, and digital health) face many barriers in implementing the technology due to the following: (1) patentability; (2) little deployment of mHealth apps and new digital tools in current clinical practices; (3) a communication gap between multiple stakeholders (which include technology entrepreneurs, investors, developers, researchers, and practicing physicians) because of the complexity and involvement of experts from diverse domains in digital health projects (the data shows that clinical experts were only involved in the implementation phase and were not represented or asked for input at the planning and initiation phases of the interventions); (4) lack of evidence on the validations of digital health devices and smartphone apps; (5) unavailability of regulatory frameworks.

On the other hand, the existing technologies currently used do not support large data sets, the replacement of gadgets is costly, and the tools for natural language processing for local languages are unavailable.

#### Catalyzing Culture

There is a communication gap when it comes to exchanging ideas and initiatives, primarily between physicians, technology experts, and researchers. Hence, there is a need for better communication to scale up and integrate digital health into the health care system in Pakistan. In addition, there should be advanced collaborative pathways to improve user wellbeing where the roles of investors, their responsibility, rights, and transparency should be clearly described. The result of this study shows that participants felt that there is a lack of guidance on how to utilize technologies to better suit the objectives of digital health interventions.

#### Workforce Training Duration

On-the-job training becomes pivotal in enabling individuals to acquire essential skills (related to technology advancement) for a meaningful contribution. For the teams involved in these projects to acquire a basic understanding before initiation, 2 to 3 training days were considered. Another important point raised was the need for digital health certificate programs for an average of 6 months, as these can advance one’s career further.

### SWOT Analysis

#### Profiles of the Projects

The set of projects selected for this study is representative of all the provinces in Pakistan. The academic sector is contributing significantly to digital health in Pakistan. There were a good number of projects which were funded internally through their parent organization; this is an important factor in increasing the chances of the scalability and sustainability of the intervention as self-reported by the participants. The interventions in these projects targeted the general health of the population, focusing on both communicable and noncommunicable diseases, and providing services to individuals from all socioeconomic groups. There was, however, less focus on subspecialty medicine in the interventions as most projects were found to revolve around family medicine and general health domains. The interventions under study did not use geospatial technology as part of their interventions for local health problems such as regional outbreaks, epidemics, and surveillance activities. The use of AI and ML was observed to be used at the rudimentary level, either due to a lack of big data sets or access to the main datasets.

A significant proportion of the interventions were based on smartphones, which are owned by less than one-third of the population [49]. Digital health is steadily growing as an industry in Pakistan, and there are a lot of opportunities to work with different health care providers in both programmatic and research domains as part of public health and clinical settings. Globally, digital health has been used to address domains like mental health and maternal and child health; however, in our SWOT analysis, there were limited studies in this domain and hence this could be a very important opportunity—perhaps even more relevant now due to the COVID-19 pandemic [50]. AI and ML are currently revolutionizing the digital health landscape across the world, and the Pakistani health care community needs to take advantage of such technologies to derive significant benefits for the population’s health.

In our study findings, one of the major constraints in conducting and scaling up technology-based interventions and programs in Pakistan was the low availability of human resources with technology-specific skills in addition to restricted local training and capacity building opportunities. There is also a low trend of sharing data publicly due to multiple reasons, as reflected in the low percentage of studies published in the public domain, an issue that raises serious concerns. The technologies being used in most projects are old generation, and this can make stability and sustainability challenging to achieve. Currently, there is a trend in Pakistan to develop custom-made software in-house, thus limiting value for money and creativity. Further details are displayed in Multimedia Appendix 2.

#### Project Team Characteristics

The teams were diverse with professionals belonging to different disciplines, reflecting collaboration across different sectors. A good representation of IT professionals is central to making sure the projects are technologically sound. The results of the study show that professionals belonging to different industries prefer project novelty to be a focus in the domains of telehealth, AI diagnostics, and digitization of EPI data. At present, the projects are in various phases, including exploratory, research, ideation, proof of concept, and implementation.

There is a noticeably low representation of individuals with research and evaluation backgrounds in the project teams, and IT experts and clinical teams might not have the bandwidth to evaluate the impact of these projects. The basics of digital health and its importance can be introduced to students at the undergraduate and postgraduate levels in health care and IT disciplines. There is room for better collaboration across various sectors. Health care and IT professionals especially have an important role to play in shaping the future of digital health and therefore need to get involved. There was a significant proportion of projects designed entirely by individual developers. While this may have certain advantages, often these developers lack the insight that health care professionals have regarding the needs of the patients and the health system in general.

#### The Technology Used in Current Projects

Most projects are employing advanced technologies to store data. MySQL is a popular, easy-to-use framework [51,52]. Most initiatives are currently saving data on local servers, which ensure data privacy. The local servers used for storage come with the drawback of high maintenance cost and high bandwidth. Also, these servers may not be practically usable when the interventions are scaled up. Projects studied are also employing interactive modes of communication to engage the users better.

Some projects are still using outdated programming frameworks that have been replaced worldwide, inhibiting the use of state-of-the-art analytical and decision support features such as Python and geospatial technology such as imaging, analytics, mapping tools, and AI and ML. These limitations can be overcome with the use of technology frameworks that consume lower bandwidth, capacity building for Python and other modern technical software languages, AI and MI, and cloud-based services. It was also noted that the majority of the end-users of the projects required some training before use. There is little to no cybersecurity and regulation of the technology used to gather the data, and little expertise to develop more secure systems. There is also a lack of an established mechanism to determine the effectiveness of training and capacity in the area of cybersecurity.

#### Project Evaluation

At the time of the survey, most of the projects were undergoing an evaluation process using a variety of tools, and a high proportion of them were being scaled up, which reflects the acceptability and success of digital health interventions in the society. Most of the participants consider the strengths of the project in terms of technology, focusing on the use of hospital information systems, AI models, patient monitoring and information tracking system, cloud base, time consultation, and square database in their existing projects. On the other hand, the participants consider a lack of resources, unavailability of technology solutions, and a lack of integration with allied systems as the major factors limiting project advancement and scalability. There is an extremely low ratio of manuscript publications in the digital health domain due to the lack of technical skills and ethical implications; hence, most of the relevant work is not published on scientific forums. Therefore, the majority of projects are only being reported on project websites and blogs, which may often exaggerate the impact of the interventions and may not be based on valid evidence or reality.

There are a lot of opportunities to employ AI and ML techniques in the evaluation of big data sets, as these tools are readily available in Pakistan. More projects need to be connected with national databases and registries. This will help in scaling up the projects from the implementation and commercialization perspective. Stringent monitoring and evaluation of the overall project, in addition to technology and clinical aspects, need to be improved. Furthermore, improvement in the reporting standards is also required to enhance the quality and efficacy of digital health-based interventions and programs. There is an increased reliance on conventional paper-based checklists and rudimentary tools lacking coverage of technology and health aspects.

#### Ethics in Digital Health

All our respondents agreed on having their digital health projects regulated by independent ethics committees, with access to data limited to relevant personnel only. The stakeholders were also mindful of the importance of data retention and archiving. Of the 51 projects examined, 10% (5/51) did not obtain consent from the end-users related to the project, which might include clinical or personal identifier data. Around half of the studies had ethical approval to implement the projects or the study. The majority of the participants felt the need for an independent ethics committee led by the local institution as opposed to a regional or public based ethical committee. However, one common critique of local ethical boards is the poor quality and the lack of adherence to national and international guidelines. Unfortunately, not following the standard guidelines might have ethical and legal implications not only for the study but to the parent institute as well. Hence, strict compliance with the ethical standards set by national and international standards is highly recommended.

There is also a pressing need at the national level to establish ethical frameworks, standardize consent norms, and identify the regulatory approvals needed before a project can be implemented and launched, especially when something is scaled at a larger level. The Ministry of Information Technology and Telecom in Pakistan appears to be the body with the relevant mandate. There is currently a limited national effort dedicated to addressing the ethical concerns that arise in digital health projects, such as data privacy, retention, and determining the situations when consent is not required.

## Discussion

### Principal Findings

This is the first study conducted in Pakistan that looks at digital health interventions at a national level using SWOT analysis, and that also examines the technology and ethical dimensions of digital health. In the baseline survey, both qualitative and quantitative data were collected using a mixed-methods strategy; therefore, SWOT analysis was the most suitable strategy to analyze the data and highlight the impact of different digital health initiatives in Pakistan, as it allowed for the identification of internal factors as well as external factors that influence the implementation of these initiatives.

Overall, digital health or the use of technology in all domains of health is an emerging factor globally, and especially in resource-constrained settings; it can be a powerful solution for improving health outcomes locally and at the grass-root levels. This study and SWOT analysis were conducted just before the global COVID-19 pandemic. However, the findings obtained resonate with the demand and improvement in digital health interventions and innovations in line with the health system. Since the COVID-19 pandemic, the role of and demand for digital health has increased significantly, and in some scenarios, it is the only viable solution for moving forward while following a social distancing strategy. In Pakistan, the use of digital health technologies in handling the COVID-19 pandemic has surged, especially in the public sector; 3 notable interventions being implemented include: (1) real-time registries and dashboards to visualize and download positive cases and relevant data, (2) a COVID-19–specific telehealth portal where patients can consult a doctor online, (3) an SMS text messaging–based EHSAAS emergency cash transfer program providing financial support to citizens identified by the government’s poverty criteria during the enforced COVID-19 lockdown [53–56].

Due to resource constraints, the current infrastructure of Pakistan still struggles with providing access to high-speed internet and smartphones to two-thirds of its population [46]. Furthermore, high budget requirements in the establishment and implementation of technology-based interventions, including human resources and infrastructure costs like software and servers, is a major barrier among countries under-budgeted for technology and health sectors such as Pakistan [53]. This limits the potential impact of technology-based interventions that could be carried out for a more extensive and socioeconomically diverse audience [46].

Our study revealed that 36% (18/51) of the current projects involve smartphones as a medium for service delivery, which is in line with Pakistan Telecommunication Authority (PTA) data which states a similar number of smartphone ownership. However, on closer inspection, most of the smartphone owners are concentrated in urban populations, sometimes with one person owning more than one smartphone [46]. Similarly, the nationwide Pakistan Demographic and Health Survey 2017-2018 revealed that 22.9% of urban households had an internet connection compared to 4.9% in rural areas [54]. While smartphones offer an interactive interface and allow for more complex web-based applications to be developed, the generalizability of these interventions or programs is limited due to the non-accessibility of smartphones to the majority of the population in present-day Pakistan. Hence, looking at the ground reality, any successful interventions with an excellent outreach would have to be generalizable to a majority of the population, even if it is not the most advanced technology.

Our SWOT analysis also highlighted that most hospitals, basic health care units, and tertiary centers do not have the capacity to incorporate digital health interventions, and therefore all the efforts being made in this area are fragmented and vertical. Electronic health records (EHRs) are an indispensable tool in aligning digital health projects with the existing health system [55,56]. A study done in the province of Khyber Pakhtunkhwa in 2017 revealed that of the 35 hospitals studied, only 1 (0.03%) fulfilled all the requirements of basic EHRs [57]. An eHealth readiness assessment in Pakistan reported low e-literacy in health care professionals and showed that the cost of technology is a major barrier faced by hospitals in introducing EHRs, reflecting the need for health care staff training on the proper usage of digital health tools and technology before technology implementation on a larger scale [61–63].

The projects in this study originated from various sectors, with equal representation from the public (17/51, 34%) and private (17/51, 34%) sectors. This is a novel approach, as previous research was less balanced (eg, in a study based in Bangladesh, the private sector was the primary contributor [44]). The active involvement of the public sector is very promising, as no sustainable impact can be made without the active participation of the public sector.

One major gap identified in the analysis was the lack of ethical and legal regulations at the national level. The Ministry of Health and the Ministry of Information Technology and Telecommunications in Pakistan have to work together to implement a policy for regulating the use of technology in the health sector and standardize the procedures for developing and executing digital health–based projects. However, health is a provincial matter in Pakistan while IT is a federal subject; this will present a challenge in this regard. Our results also showed that only 1 project was connected to a national database; this demonstrates the need for better collaboration and communication between the different stakeholders and the government toward sharing the data and unifying the currently fragmented system. Another barrier related to academia and digital health was the absence of undergraduate and postgraduate level degrees and courses in Pakistan. Several of the weaknesses and threats associated with human resource capacity are linked to the lack of any formal program in digital health in Pakistan. Lastly, as per our SWOT analysis findings, the main risks associated are (1) data storage and retention; (2) hidden costs due to storage, security, processing, archiving, analyzing, and cloud; (3) unorganized dataset leads to incompetent analytics and faulty trends and predictions; and (4) data privacy and security.

### Strengths and Contribution of the Study

To the best of the authors’ knowledge, this is the first study that looks into digital health interventions in Pakistan as a whole. Rather than just considering the PubMed published literature, the stakeholders with both published and unpublished projects were interviewed for the SWOT analysis to provide a more holistic picture of current digital health-related projects.

### Limitations

This study was conducted by researchers from academic universities; thus, there is a possibility of low representation of projects from industry and public domains. In addition, only the interventions conducted in the last 5 years were taken into consideration, and the work done in this field before that might have been missed due to possible recall bias. The authors approached all potential stakeholders, but consent was denied in a few cases. The respondents self-reported whether their projects are or have been evaluated; however, this could not be verified by an independent source. Lastly, since digital health is a relatively new field in Pakistan, there are still some grey areas as to what interventions fall in the domain of digital health; therefore, some relevant work may have been missed. However, the investigators have tried their best to include all the interventions carried out in Pakistan in the domain of digital health in the last 5 years.

### Conclusions

Digital health–based interventions are, slowly but steadily, being ushered into the existing health system of Pakistan. There are still significant hurdles, barriers, and roadblocks in the form of limited internet facilities, phone ownership, network coverage, unavailability of regulatory frameworks, data protection and security regulation, accessibility, affordability, and paper-based health records, limiting the types of technologies that can be utilized for effective interventions. However, despite all the challenges, digital health is steadily expanding through the efforts of multiple stakeholders in both the public and private sectors. It is difficult to say how effective these interventions have been, as not all interventions are being evaluated or published. The future for digital health does look bright, especially after the government’s new initiative to digitalize the public sector in Pakistan [58].

## Appendix

Multimedia Appendix 1 Resource list: details of all the projects, studies, and other grey literature considered for the strengths, weaknesses, opportunities, and threats (SWOT) analysis.

Multimedia Appendix 2 Strengths, weaknesses, opportunities, and threats (SWOT) analysis table.

## Abbreviations

AI: artificial intelligence
COVID-19: coronavirus disease 2019
DHIS 2: District Health Information Software 2
eHAP: eHealth Association of Pakistan
EPI: expanded program on immunization
HIV: human immunodeficiency virus
IT: information technology
LMICs: low and middle-income countries
MeSH: medical subject headings
mHealth: mobile health
ML: machine learning
NCDs: noncommunicable diseases
NHS: National Health Service
PostgreSQL: Postgres Structure Query Language
PTA: Pakistan Telecommunication Authority
SQLite: Structured Query Language
SWOT: strengths, weaknesses, opportunities, and threats
WHO: World Health Organization
