# Supplementary material for: Current Challenges of Digital Health Interventions in Pakistan: Mixed Methods Analysis
Source: J Med Internet Res. 2020 Sep 3;22(9):e21691. doi: 10.2196/21691 (PMC7499163; doi:10.2196/21691)
Supplement: Multimedia Appendix 2 [file jmir_v22i9e21691_app2.pdf]

SWOT analysis table

This is a Multimedia Appendix to a full manuscript published in the J Med Internet Res. For full copyright and citation information see http://dx.doi.org/10.2196/jmir.21691"

	Strengths	Weaknesses	Opportunities	Threats
**Project Details**	• Representation of all provinces<br>• Good representation from academia signifies innovation<br>• A higher proportion of internal and international funding as compared to national funding<br>• More internal funding shows interest from the sustainability perspective<br>• More focus on research and implementation n showcases competitiveness<br>• More representation from family medicine domain<br>• More digital health-based interventions focusing on noncommunicable diseases<br>• More interventions targeted at the general population as opposed to any particular age group	• System development not at par with research focus which can impact scalability and sustainability<br>• The low representation of specialized field as compared to general health/family medicine.<br>• Lack of use of geospatial technology and analysis.<br>• Lack of big data sets hence resulting in less efficient data mining machine learning and artificial intelligence models.<br>• Two-thirds (66%) of the Pakistani population has functional phones, yet the majority of the interventions were smartphone based<br>• Lack of skilled human resources for digital health	• A recent influx of digital health interventions in Pakistan in both implementation and research domain<br>• Capacity building opportunities for application system developers<br>• Opportunity for open source applications for high accessibility of digital health based interventions<br>• The higher opportunity of digital health working with different health domains, both pragmatic and research.<br>• Focus on neglected health outcomes like mental health.<br>• Higher opportunity to work in maternal newborn and child health<br>• Replication of successful digital health projects from other countries.<br>• More projects focusing on artificial intelligence/ machine learning models.<br>• Merging diverse Digital health components can provide more effective results	• Unwillingness towards sharing data for impact maximization<br>• Most projects reflecting older/conventional technologies thus risking sustainability and scalability issues<br>• Higher in-house development limits value addition and impact<br>• More international funding may generate copyrights/IP issues<br>• Scarcity of funding<br>• Low conversion of digital health-based interventions for scale-up and commercialization.<br>• No policy for geospatial mapping at national level<br>• Lack of a clear understanding of digital health among key stakeholders.<br>• The high cost of digital health-based interventions.<br>• Lack of availability of data related to digital health interventions at national and provincial levels.<br>• Lack of infrastructure in basic/ primary health units and tertiary centers to

SWOT analysis table

		• Low mobile phone network coverage in some areas		implement digital health interventions.

SWOT analysis table

	• Balanced coverage of Interventions from all Socioeconomic classes<br>• Application domains comparable to international trends showcase the relevance<br>• Balanced representation of both technology hardware and software in digital health interventions<br>• Increased technology interventions over time<br>• Participant recruitment is more straight forward in Pakistan and thus not identified as a barrier<br>• Scalability at the provincial /national level in most projects<br>• High ratio of scale-up of completed projects<br><br>•Availability of technology infrastructure to scale digital health interventions Adequate resource allocations	• Absence of national digital health framework	and high impact (interoperability)	

SWOT analysis table

	(costs) to various intervention components<br>• Presence of more open source applications			

**Project Team Details**	• Diversity among professionals	• Less representation of regularized interventions	• Students' involvement can make them career-oriented	• Individual developers-led projects may lack the insight of a health care professional regarding healthcare needs and challenges and vice versa

SWOT analysis table

	• Reflects better collaboration The abundance of IT experts ensures intervention is technologically sound and has excellent reliability.	shows gaps at the governance level .<br><br>• The low representation of individuals with a research background in the team, IT experts and clinicians may not have the skills to evaluate the impact of the project	• Towards digital health, medical, and engineering.<br><br>• The curriculum should teach digital health and its significance Collaboration across sectors to utilize each other's expertise – including Academia, pragmatic and industry<br><br>•More involvement professional from both Healthcare and Engineering in Digital health	
**Project Evaluation**	• A higher proportion of evaluation of studies/ projects for digital health<br>• A high proportion of projects at scale up stage	• Data not reported or published at national or international forums	• Availability of relevant tools for study evaluations including big data.<br>• Application of artificial intelligence/ machine learning in future digital health projects Installation and setup of app/project to a national database	• Reliance on conventional tools<br>• Restricted access to projects to the national database<br>• Risks arising from lack of structured programs/certifications for managing large data sets<br>• Low evidence-based evaluation of digital health interventions

SWOT analysis table

			• Commercialization / generalization of scale-up projects Stringent monitoring and reporting	
**Ethics in Digital health**	• Responsiveness s towards ethical requirements<br>• Data access restricted to relevant personnel<br>• Mindfulness towards data retention and archiving.	• Around 1/10th (10%) of the studies/project s didn't administer consent<br><br>• The low representation of government regulators within regional ethical committees.	• Standardization of consent forms and procedure<br>• The national body regulating ethical challenges related to digital health<br>• Develop regulations for formally initiating digital health projects.	• Lack of proper ethical regulatory framework to determine if consent is required or not and data retention policy<br>• Local institute committee can influence/bias ethical decisions
